# Aro: a machine learning approach to identifying single molecules and estimating classification error in fluorescence microscopy images

**DOI:** 10.1186/s12859-015-0534-z

**Published:** 2015-03-27

**Authors:** Allison Chia-Yi Wu, Scott A Rifkin

**Affiliations:** 1 Graduate Program in Bioinformatics and Systems Biology, University of California, La Jolla, San Diego, CA USA; 2 Section of Ecology, Behavior, and Evolution, Division of Biology, University of California, La Jolla, San Diego, CA USA

**Keywords:** Single molecule imaging, smFISH, Random forest, Image informatics, RNA, Fluorescence microscopy, Machine learning, Image quality

## Abstract

**Background:**

Recent techniques for tagging and visualizing single molecules in fixed or living organisms and cell lines have been revolutionizing our understanding of the spatial and temporal dynamics of fundamental biological processes. However, fluorescence microscopy images are often noisy, and it can be difficult to distinguish a fluorescently labeled single molecule from background speckle.

**Results:**

We present a computational pipeline to distinguish the true signal of fluorescently labeled molecules from background fluorescence and noise. We test our technique using the challenging case of wide-field, epifluorescence microscope image stacks from single molecule fluorescence *in situ* experiments on nematode embryos where there can be substantial out-of-focus light and structured noise. The software recognizes and classifies individual mRNA spots by measuring several features of local intensity maxima and classifying them with a supervised random forest classifier. A key innovation of this software is that, by estimating the probability that each local maximum is a true spot in a statistically principled way, it makes it possible to estimate the error introduced by image classification. This can be used to assess the quality of the data and to estimate a confidence interval for the molecule count estimate, all of which are important for quantitative interpretations of the results of single-molecule experiments.

**Conclusions:**

The software classifies spots in these images well, with >95% AUROC on realistic artificial data and outperforms other commonly used techniques on challenging real data. Its interval estimates provide a unique measure of the quality of an image and confidence in the classification.

**Electronic supplementary material:**

The online version of this article (doi:10.1186/s12859-015-0534-z) contains supplementary material, which is available to authorized users.

## Background

In the last decade, a host of new technologies for tagging and visualizing individual molecules have yielded unprecedented quantitative insight into the spatial and temporal dynamics of fundamental biological processes as varied as ligand-receptor interactions at the cell surface [1], protein localization to synaptic junctions [2], and incomplete penetrance [3]. For example, the ability to visualize mRNA transcripts at the single molecule level without transgenic methods has led single-molecule fluorescence *in situ* hybridization (smFISH) to be widely used in studying gene expression in various organisms [3-14]. Recently this technique has been pushed to image up to 32 genes simultaneously with the promise of increasing this number still more [11]. These microscopy-based techniques rely primarily on fluorescent proteins or dyes that are bound to the molecule of interest and appear as a bright, roughly Gaussian spot. Background fluorescence can be considerable for some of these techniques, including smFISH [4,8], which makes distinguishing signal from noise an image processing challenge. However, a statistically principled, automated, and robust method for analyzing the images and classifying local intensity maxima as signal or noise, and estimating the accuracy and variability of these classifications has not been developed. This problem is acute since highly sensitive microscopy methods like smFISH are ideally suited for quantitatively studying stochastic variation in gene expression and other molecular processes within a population.

We have extended a machine-learning pipeline for identifying, localizing, and counting biologically meaningful intensity maxima in 3D image stacks [15] both by improving the initial spot classification and, crucially, by providing a way to both estimate the quality of the data and generate an interval estimate for the number of molecules in it. We have tested it extensively on the challenging case of wide-field epifluorescence smFISH image stacks of nematode embryos where there can be substantial background fluorescence, and it also works on other samples like yeast and mammalian cell culture where the signal to noise ratio is more favorable. Unlike other commonly used methods [3,16,17], this software does not rely on arbitrary or user-defined parameters and cutoffs, but instead recognizes and classifies individual mRNA spots by measuring several features of local intensity maxima and classifying them with a supervised random forest classifier [18,19], It is a spot-centric approach as compared with approaches that involve thresholding an entire image [3,16,17].

## Implementation

**Figure 1:**
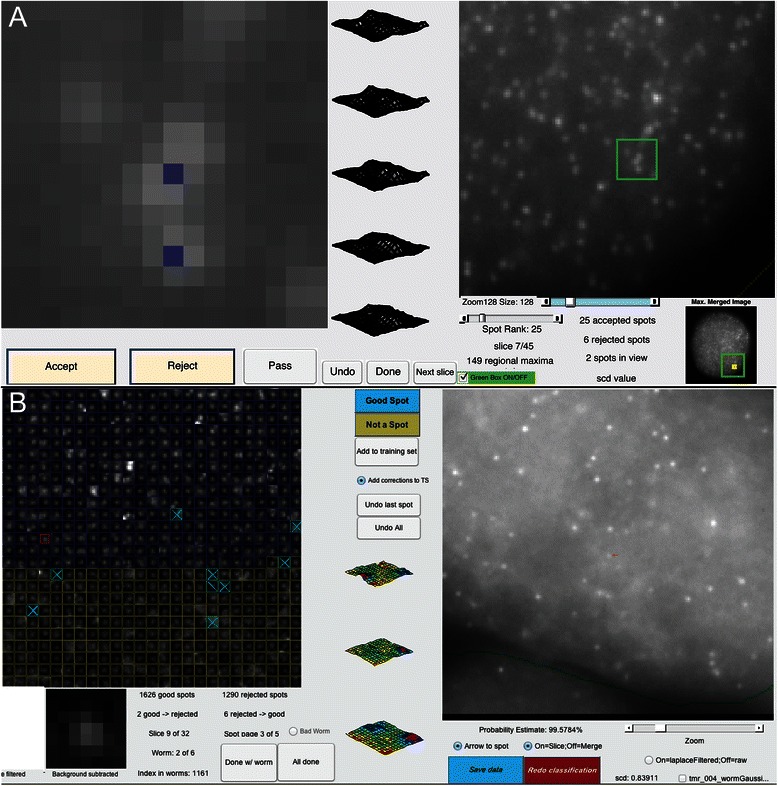
**GUIs in Aro. A**. The training GUI. The left plot is a 16 × 16 square of pixels from the image on the right with local maxima (candidate spots) marked in blue. The user has the option to designate the maxima as signal, noise and add them to the training set or to skip them. **B**. The reviewing GUI. The left plot is a grid containing each identified local maximum ranked by its vote. Blue outlines mark signal spots; yellow boxes mark noise spots. The user has the option to correct classifications and retrain the classifier. The image on the right shows the context for the spot currently in focus (red outline).

Machine learning has been remarkably successful in a variety of classification and prediction tasks [20,21]. As with all supervised machine learning techniques, our pipeline trains a classifier based on a curated training set and then applies this classifier to new data. Our implementation includes a GUI to create the training set and a GUI for review and revision of the final classification (Figure 1). This review GUI also allows the user to retrain the classifier incorporating any corrections. (If a dataset only consists of a few image stacks, the GUIs used for either the training or review could be used to manually curate the images without the need for machine learning). The software currently uses the random-forest implementation provided in the MATLAB Statistics Toolbox [18].

**Figure 2:**
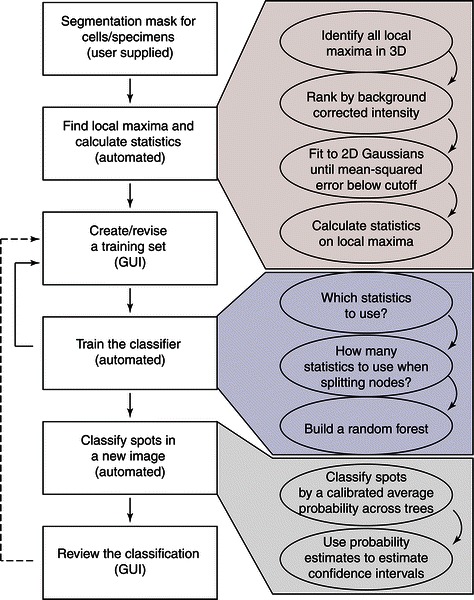
**Flowchart of the analysis pipeline with details of the automated steps.**

The first step for processing either a training image or a new image is to identify all local intensity maxima (spots) within an image stack and rank them in descending order by their background corrected intensities (Figure 2). These are then sequentially fit to 2D Gaussian surfaces until the mean squared error from the fits are persistently less than a cutoff value below which local maxima are empirically found never to be true signal spots. This cutoff is set to be extremely conservative because its function is simply to save time and memory by removing the majority of local maxima that represent noise in an image stack. The heart of the pipeline is a random forest classifier [19] – an ensemble of decision trees built from bootstrapped training sets – which has been shown to produce highly accurate classifications in a wide variety of applications [22-29].

Our training GUI allows a user to view spots from a subset of image stacks in the dataset, generate a manually curated training set by classifying them as true signal spots or noise, and build a forest of decision trees based on the features calculated from each spot (Figure 1, Additional file 1). We find that a training set consisting of a few hundred positive and negative examples is sufficient for stable classification. For each tree, the algorithm selects a bootstrapped sample from this training data. Each split in the tree is based on a randomly chosen subset of the statistics, and the tree is grown according to pre-specified stopping criteria. The leaves can be, but are not necessarily, comprised of a single class. At the end of training, the user has a bagged ensemble of decision trees.

To classify a new local maximum, the program runs the statistics for the putative spot through each tree to a terminal leaf. The proportion of training spots in this leaf that are manually classified as good can be used to estimate the probability that the new local maximum is a true signal spot. Although such probabilities are known to be inaccurate for single decision trees [30], using an ensemble of bagged trees improves the probability estimate, and so we average these proportions for a single candidate spot across all the trees in the forest to estimate a *preliminary* probability that it is a true spot [30-33]. However, these preliminary probabilities do not necessarily reflect the long-run frequency of a spot with particular features being classified as signal or noise [34-36].

**Figure 3:**
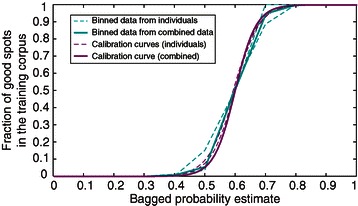
**Calibration curves based on bagged probability estimates are robust to individual curation differences.** A large corpus of manually curated training spots was binned by the bagged probability estimate derived from averaging the probability estimates for a local maximum from each tree in the ensemble. The calibration curves are constructed by fitting a sigmoidal function to the plot of bin centers (0 to 1 by steps of 0.1) versus the proportion of curated good spots in that bin. Calibration curves based on bagged probability estimates are less susceptible to curation differences between individuals than ones constructed by majority rules voting (Additional file 2).

In order to calibrate these preliminary probability estimates and transform them into more accurate probabilities, we use empirical data derived from thousands of training spot examples curated by different people. We bin this data by the preliminary probability estimate and then count the number of true spots in each bin. We fit a sigmoidal function [36,37] to the plot of the proportion of true spots against the probability estimate, and we use this function to transform the preliminary probability estimate of a local maxima being a true spot (derived from averaging across the decision trees) to an empirical probability estimate based on curated data (Figure 3, Additional file 2). This calibration curve is remarkably similar for different users and different datasets, and users can create their own calibration curves based on their own training sets. If the calibrated probability is greater than 50%, the local maximum is classified as a true spot. The user can then review and edit the classification using the review GUI and has the option to add any corrections to the training set, re-train the random forest, and re-run the classification (Figure 1). At any time the user can remake the random forest based on the augmented training set and rerun the classification.

The calibrated probability reflects uncertainty in the classification of any particular spot, and, consequently, can be used to measure the uncertainty in the count of the number of true spots in an image. A local intensity maximum with a particular preliminary probability can be thought of as a sample of size 1 from the population of all candidate spots with the same preliminary probability, of which some fraction (the calibrated probability) are true spots. We would like to estimate a confidence interval for the count of true spots in an image. The width of the confidence interval is a measure of the quality of an image because it will largely be driven by the fraction of spots for which the user himself or herself would be ambivalent, based on how he or she has classified similar spots in the training set.

To construct the interval estimate. we conduct a set of *n* Bernoulli trials with variable probabilities (also known as Poisson trials) [38] where *n* is the number of local maxima tested in an image. The variable probabilities (*p*_*k*_) are based on the calibrated probability estimates for each local maximum (indexed by *k*) (see Additional file 1 for details). The number of good outcomes (*X*_*k*_ = 1) in this set of Poisson trials is a simulated estimate for the number of transcripts in the image.

$$ T\sim \left[{\displaystyle \sum_{k=1}^n}\left({X}_k\left|\;P\left\{{X}_k=1\right\}={p}_k\right.,\;P\left\{{X}_k=0\right\}=\left(1-{p}_k\right)\right)\right] $$

By rerunning this model 1000 times, we can derive a confidence interval for the total spot number (*T*). This interval will be tight for high quality images and will widen as image quality degrades.

Estimating the variance of random forest and other bagged predictions is still an open problem, in part because the variance is comprised of both (a) sampling variance from training on a limited set of data and (b) Monte Carlo effects arising from a finite amount of bootstrapping [39-41]. Because we can empirically calibrate random forest probabilities in our classification task, we can take advantage of standard probability theory to construct an interval estimate.

## Results and discussion

One key difficulty with evaluating image-based, molecule counting methods is that there is not an independent way to count the number of molecules in the specimen. For smFISH (as well as other techniques) it has been experimentally established [4,8] that, with the exception of transcriptional foci, spots in these images do represent single, fluorescently-labeled, diffraction-limited molecules. We can, however, use artificially generated data to investigate how well our method performs in the face of background noise.

In order to avoid making arbitrary assumptions about the structure of background noise, we used three 3D image stacks from actual specimens without any transcripts as the background. The background therefore consists of both autofluorescence and any diffuse fluorescence due to unbound probes that were not removed by washing. To generate signal, we sprinkled point sources of a specified magnitude throughout a blank image stack of the same size as the background stack, convolved them with a point spread function based on typical microscopy parameters, added the background and signal stacks together, and then blurred them with a Gaussian filter. The spots in these images look very much like actual data (Additional files 3, 4 and 5).

**Figure 4:**
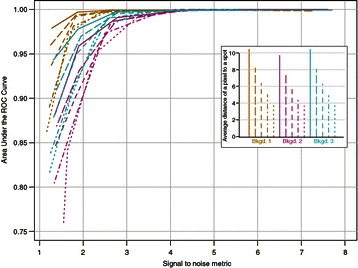
**The algorithm performs well even as data quality degrades.** We tested the software on artificially constructed images of varying quality with realistic signal intensities, spot densities, and background noise based on real data. Spot density is measured by average distance of a pixel to an artificial spot in an image and is shown in the inset. Signal intensity on the x-axis is the average pixel intensity at the centers of the random spots minus the mean pixel intensity of the image divided by the standard deviation of the pixel intensities in the image. A sample of spots from the background 1 images were used to train the classifier.

To test our method, we used artificial images based on one background to construct random forests and evaluated the false positive and false negative rates for the images based on the other two backgrounds. The method performed robustly (Figure 4), with the area under the ROC curve well above 90% for realistic signal intensities and spot densities (Figure 4, Additional files 3, 4 and 5). The width of the confidence intervals increased at lower signal to noise levels, but otherwise was a fairly constant fraction of the total spot count. The software can reliably distinguish spots that touch, particularly if the local intensity maxima are separated by at least two intervening pixels. However, when the local mRNA density is too high, it is even impossible for humans to distinguish individual spots. Under these circumstances an intensity and regression-based approach to estimating transcript levels, while noisy, may be the only option [11,42].

**Figure 5:**
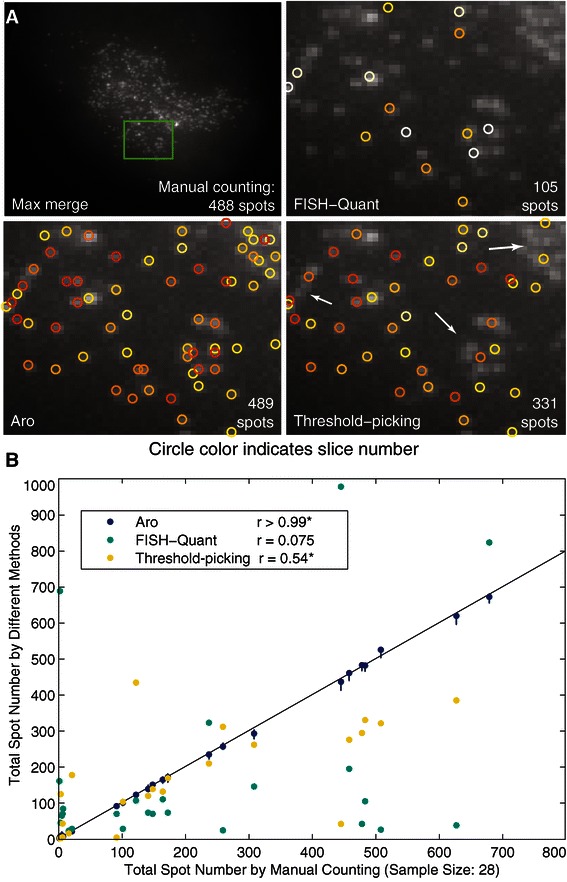
**Comparison of spot identification and classification methods. A**. The upper left is a maximum merge projection of an smFISH image from a *C. elegans* embryo for which 488 signal spots were counted by hand (using the GUIs described here). A green rectangle highlights a section of the image. The other three images show spots identified in this green rectangle by FISH-Quant (upper right), the threshold-picking method (lower right), and the method described here (Aro: lower left). The number of signal spots identified in the embryo by the various methods are noted in the lower right of each image. Circles mark the locations of identified spots and are color coded by z-slice. Arrows point to representative areas depicting the tendency of threshold method to identify a large high intensity region comprised of several spots as a single spot. **B**. A plot of manually counted spot number (x-axis) and estimated spot number (y-axis) by Aro, threshold-picking, and FISH-Quant across 28 *C. elegans* embryos. Both FISH-Quant and threshold-picking tend to underestimate the true number of spots (particularly at higher spot counts for the threshold method) while our Aro machine learning method performs well across a range of spots numbers. Spearman correlations (r) between the true and estimated spot number are listed for each method. Both Aro and threshold-picking perform significantly better than random on this dataset. Interval estimates are depicted for Aro. Neither FISH-Quant nor threshold-picking provides a way to estimate error.

A few unsupervised algorithms have been used to automatically count the number of spots in smFISH [3,16,17,43-45]. Two of them [3,16] use a watershed method based on intensity to find a range of intensities over which the number of connected components in the image is insensitive to intensity thresholding. This number is taken as an estimate of the spot number. However, when the expression level is high, spots are often clustered, and out-of-focus light gives a higher local background that can vary across an image. Under these common circumstances these methods underestimate the true signal (often because neighboring spots are lumped together as one) while the method described here performs consistently well with few or many spots in the image, even if they touch (see above) (Figures 3 and 5, Additional files 3, 4 and 5). FISH-Quant [16] further analyzes the connected components, but its performance can be very sensitive to user-defined global parameters when the background signal is high (Figure 5).

Another approach [43-45] has the primary goal of spot localization and starts by identifying individual candidate spots after intensity thresholding a 2D maximum projection, correcting for local background, and fitting them to 2D Gaussians. It then removes purported duplicates and thresholds a measure of the intensity of the entire spot to distinguish signal from noise. Because our algorithm also starts directly from the local maxima, it also works robustly for images with high or inhomogeneous backgrounds. However, it uses the 3D image, not a maximum projection, and is able to resolve clustered spots. Furthermore, while our local-maxima-centric approach uses a similar method for localization, its primary goal is robust classification without setting semi-arbitrary thresholds. The supervised learning process and the GUI allow the user to manually curate the classification of individual spots, and then feed these corrections back into the classification algorithm. This is particularly useful for low quality images, allowing the user to overrule the algorithm for spots on the boundary between signal and noise.

## Conclusions

As the throughput of microscopy-based single-molecule techniques increases, robust image processing techniques will be ever more crucial. We present a machine-learning-based pipeline for identifying and classifying fluorescently labeled molecules in 3D image stacks that performs well under conditions where other algorithms fail. The software (called Aro Spot Finding Suite after *Arothron hispidus*) includes MATLAB [18] GUIs to generate the training set and review the classifications and a detailed manual with examples. The ability to infer biological meaning from a quantitative imaging experiment depends upon extracting reliable measurements from images. For single molecule imaging, our software uniquely provides a way to measure this reliability.

### Availability and requirements

**Project name**: Aro Spot Finding Suite.

**Project home page**: https://gitlab.com/evodevosys/AroSpotFindingSuite.

**Operating system**: Platform independent.

**Programming language:** MATLAB.

**Other requirements**: MATLAB statistical toolbox; third-party MATLAB packages that are included with their own licenses with this distribution.

**License:** Apache 2.0.

## Additional files

Additional file 1:
**Supplementary text.** Additional information about the statistics used in the classifier, the construction of the artificial data, and the user experience.
Additional file 2: Figure S1. Calibration curves based on majority rules classification are not as robust to individual curation differences as those based on bagged probability estimates. As in Figure 3, a corpus of manually curated spots was used to construct calibration curves. Instead of the average probability across trees (as in Figure 3), the initial probability estimate (x-axis) is the fraction of trees classifying a local maximum as a spot.
Additional file 3: Figure S2. Artificial data used to test the method. Maximum merge images of the image stacks constructed to test the method. One of the three embryo background used for the artificial data. The point source intensity decreases from left to right and density increases from top to bottom. The density is measured by the mean distance of a pixel to an artificial spot center. The signal to noise metric (numbers in white to the bottom left of each embryo) is the average pixel intensity at the centers of these artificial spots minus the mean pixel intensity of the image divided by the standard deviation of the pixel intensities in the image. (see Additional file 1 for details of construction).
Additional file 4: Figure S3. The second of the three embryo background used for the artificial data. The description is the same as for Additional file 3.
Additional file 5: Figure S4. The third of the three embryo background used for the artificial data. The description is the same as for Additional file 3.
