# Erratum to: ‘Aro: a machine learning approach to identifying single molecules and estimating classification error in fluorescence microscopy images’

**DOI:** 10.1186/s12859-016-1049-y

**Published:** 2016-05-09

**Authors:** Allison Chia-Yi Wu, Scott A. Rifkin

**Affiliations:** Graduate Program in Bioinformatics and Systems Biology, University of California, San Diego, La Jolla, CA USA; Section of Ecology, Behavior, and Evolution, Division of Biology, University of California, San Diego, La Jolla, CA USA

Unfortunately, the original version of this article [1] contained an error which is detailed below.

We had compared Aro to two published methods for identifying smFISH transcripts – threshold-picking [2] and FISH-quant [3]. The authors of FISH-quant were able to demonstrate that FISH-quant can perform substantially better than we were able to show. A revised fig. 5b (Fig. 1) shows the new FISH-quant results in green. Although it undercounts at high spot numbers compared to manual curation, it is far more reliable than we had shown, and any undercounting could be straightforwardly corrected.

**Figure 5b (below). Comparison of spot identification and classification methods.** B. A plot of manually counted spot number (x-axis) and estimated spot number (y-axis) by Aro, threshold-picking, and FISH-Quant across 28 *C. elegans* embryos. Both FISH-Quant and threshold-picking tend to underestimate the true number of spots (particularly at higher spot counts) while our Aro machine learning method performs well across a range of spots numbers. Spearman correlations (r) between the true and estimated spot number are listed for each method. All three techniques perform significantly better than random on this dataset. Aro and FISH-quant results are highly correlated with the manual count, and FISH-quant undercounting could be easily corrected by an appropriate factor. Interval estimates are depicted for Aro. Neither FISH-Quant nor threshold-picking provides a way to estimate error.

**Fig. 1:**
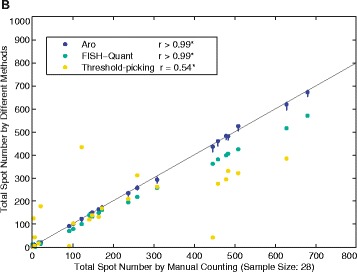
Comparison of spot identification and classification methods
