# Supplementary material for: Aro: a machine learning approach to identifying single molecules and estimating classification error in fluorescence microscopy images
Source: BMC Bioinformatics. 2015 Mar 27;16:102. doi: 10.1186/s12859-015-0534-z (PMC4450985; doi:10.1186/s12859-015-0534-z)
Supplement: Additional file 1: — Supplementary text. Additional information about the statistics used in the classifier, the construction of the artificial data, and the user experience. [file 12859_2015_534_MOESM1_ESM.pdf]

Aro: a machine learning approach to identifying single molecules and estimating classification error in fluorescence microscopy images

Allison Chia-Yi Wu and Scott A. Rifkin

Supplementary Text

Statistics (features) used for machine learning

All 2D statistics are based on a 7x7 square centered on the local maximum.

Statistics based on a 2D Gaussian fit to the spot

These statistics use http://www.mathworks.com/matlabcentral/fileexchange/31485-auto-gaussian-gabor-surface-fit/content/autoGaussianSurf.m to fit a 2D Gaussian to a spot

$$b + A\exp\left[-\left(\frac{(x-x_0)^2}{2\sigma_x^2} + \frac{(y-y_0)^2}{2\sigma_y^2}\right)\right]$$

intensity. A.

totalHeight: b+A

estimatedFloor: b

ratioSigmaXY: σ_x / σ_y or its reciprocal, whichever is smaller

The floor (b) is then subtracted from both the actual data and the fit, and they are both divided by A. Several goodness of fit statistics are then calculated using: http://www.mathworks.com/matlabcentral/fileexchange/7968-goodness-of-fit/content/gfit.m

scnmse: mean squared error between the scaled data and scaled fit divided by the variance of the scaled data

scnrmse: square root of *scnmse*

scr: correlation coefficient. correlation between the scaled data and the scaled fit

scd: coefficient of determination. fraction of variance in the scaled data explained by the scaled fit (scr^2)

sce: coefficient of efficiency. A measure of how much better the scaled fit is to the scaled data than mean of the scaled data.

Statistics based on percentiles of the intensities within the 7x7 square

prctile_X: *X* is 50, 60, 70, 80, 90. The value of the pixel at the Xth percentiles

cumSumPrctileX: *X* is 30, 50, 70, 90. Scale the data such that the minimum pixel value is 0 and the maximum is 1. Sort the scaled pixel values from lowest to highest and

compute a vector with the cumulative sum of these values. Find the Xth percentile of this cumulative sum vector.

<u>Statistics based on comparing the percentiles of actual intensities to percentiles from random values</u>

totalAreaRandPValue: pick 49 uniformly distributed numbers between 0 and 1 and sum them. Repeat 1,000 times and count the fraction of times that the sum is greater than the sum of the scaled actual data.

cumSumPrctileXRP: X is 30, 50, 70, 90. Using the same 1,000 random datasets as *totalAreaRandPValue*, compute the percentiles of the cumulative sum vector as in *cumSumPrctileX*. Count the fraction of times the percentile from the random data is larger than the percentile from the scaled actual data.

<u>Statistics based on patterns of the 7x7 square</u>

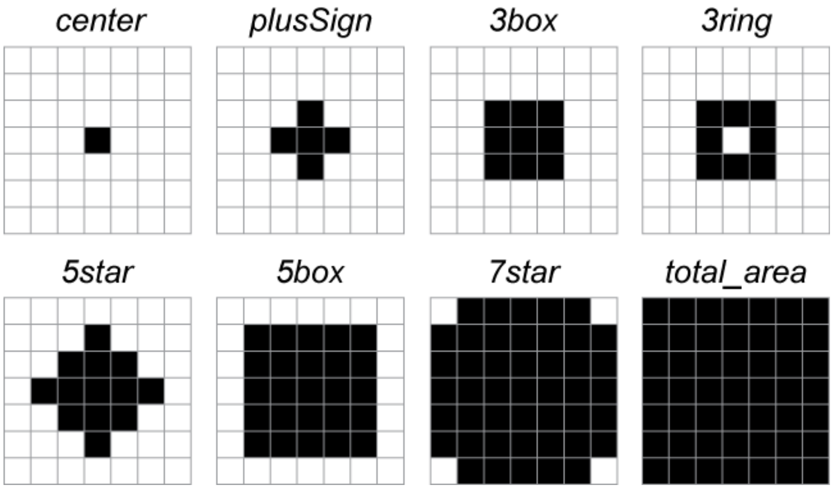

raw_X: the raw value of the black pixels in a pattern (*X*) above (except for total area)

fraction_X: raw_X / total_area

<u>Statistics based on comparing patterns of the 7x7 square to patterns where the values are permuted</u>

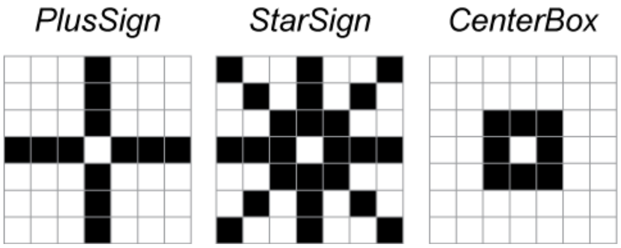

absDeltaX: fix the center pixel in the 7x7 square and randomly permute the pixel values of the remaining 48. For each black pixel, take the absolute value of the difference between the actual data at that pixel and the permuted data at that pixel and divide this

by the value of the actual data at that pixel. Sum these scaled absolute differences. Repeat this process 1,000 times and take the median of the sums.

deltaX: Same as *absDeltaX* except do not take the absolute value.

absXDelta: Sum the black pixels for the actual data and the black pixels of the permuted data. Then take the absolute value of the difference between these sums. Repeat this process 1,000 times and take the median.

XPValue: The fraction of the 1,000 permuted data where the sum of the black pixels in the permuted data is larger than the sum of the black pixels in the actual data.

<u>Statistics based on singular value decomposition</u>

svX: *X* is 1,2,3,4,5. Take each of the *m* spots in a training set and unravel its 7x7 square into a vector of length 49. This generates a matrix of size *m* x 49. Take the singular value decomposition of this matrix. Project each spot's vector onto the Xth singular vector and take its magnitude along that vector direction. The singular value decomposition is generated from the training set data. Local maxima to be evaluated are projected onto these singular vectors.

<u>Importance of different statistics</u>

While these statistics all capture aspects of what a true spot looks like, not all are equally important. When preparing to construct a random forest, the software does a prescreen to estimate the importance of the different statistics and to set aside variables that do not help to distinguish good from bad spots. These importance estimates are saved with the training set. The most important statistics tend to be the star and center box shapes and those that quantify the intensity of the spot – either total intensity or peak value – although the importance of the statistics depends upon the particular data.

<u>Generation of simulated data for validation</u>

We generated simulated images in two parts. (1) *Background*. In order to create a realistic background, we used actual images with no transcripts. We searched our corpus of data for late stage *C. elegans* embryos that were stained for a gene that is only expressed early in embryogenesis and visually confirmed the lack of identifiable signal spots. We used three such embryos as background. This avoids the need to assume a particular structure of background noise and instead uses background that consists of both unhybridized labeled oligos and autofluorescence, as does real data. (2) *Signal*. We tested a range of spot densities and spot signal intensities. For a given density we chose a set of random 3D locations in the image stack and placed the point sources at these locations. The z positions were not restricted to the actual z values of the imaged slices – they could be between slices. We convolved the point sources with a point spread function derived from our microscopy imaging setup. The point spread functions for all the point sources were then sampled at the imaged slices, and these intensities were added to the background image. We found that blurring the resulting image with a Gaussian filter of diameter 7 resulted in spots that resembled real data. Because of differences in local background and differences in the random location in z of the point source, this resulted in spots with intensities that varied.

We calculated the signal-to-noise metric of an image as the average pixel intensity at the centers of these random spots minus the mean pixel intensity of the image divided by the standard deviation of the pixel intensities in the image.

This simulated validation set consisted in total of 3 (backgrounds) x 6 (signal levels) x 5 (densities) = 90 different simulated images (Supp. figure 1). We generated a training set based on images from background #1 and used the other two background image sets as test sets. The training set contained 1200 spots taken from each of the simulated images with relatively more from the low signal level images. When making an actual training set, it is important to include an ample number of spots that are the border of signal and noise. The training set was balanced between positive and negative examples in our simulation, but as long as there are a few hundred examples of each, balance is not essential.

Variable probabilities for interval estimation

The preliminary probability of being a true spot for a local maximum is the mean of the estimated probability from each tree. This in turn is simply the proportion of true spots in the training set that are contained in the leaf of a tree. Because the preliminary probability is a mean across a sample of trees, it has some sampling variability associated with it. To propagate this variability into the interval estimate, instead of using the calibrated preliminary probability as p_k in equation 1, we tested drawing from a sampling distribution of the mean (preliminary) probability by using a normal distribution centered around the preliminary probability with standard deviation equal to the standard error of this mean. We then calibrated this number and used that as p_k. In practice, 1,000 trees gave a very small standard error of the mean (maximum ~0.013), and so this propagation has negligible effect.

User experience

As shown in figure 2, the software has both automated and GUI-based components. The processing time of the automated portion depends upon the size of the images and computational power of the user. Creating a training set takes approximately 1-2 seconds per spot for a trained user and so depends on the number of spots desired for the training set. We recommend starting with at least 100 good and 100 bad spots. This will increase when the initial classifications are reviewed. Training the classifier takes approximately 5 minutes on a 2.26 GHz Intel Xeon processor, with most of that time spent in automatically selecting a subset of features to use for classification. Classification of spots in a new image depends on the number of candidate local maxima, but is on the order of 0.05 – 0.1 seconds per candidate.

We tend to use a handful of images in a dataset to construct the initial training set and then do the initial classification on a few other randomly chosen ones. We then use the reviewing GUI to check the classification. It is often the case that the initial training set will have an overabundance of easily classified local maxima and not enough difficult marginal cases. However, it is important to have borderline cases well represented. Reviewing the initial classifications is a good opportunity to augment the training set with more difficult spots since the GUI arranges the spots in order of their calibrated probability and also shades them by their classification. Unless the initial training set included a high proportion of marginal spots, the initial classification will likely not appropriately delineate the boundary between true spots and noise. There is a tradeoff between not having a big enough training set and overfitting the training set images.

In principle, a single training set could be used for any data collected under similar imaging and experimental conditions. In practice, we tend to be conservative and make a separate training set for each imaging run, which typically consists of up to 200 images from specimens processed at the same time.
